# Isavuconazole for invasive mold disease in patients with hematological malignancies: a multicenter real-world study from China on efficacy, safety, and competing risks

**DOI:** 10.1128/aac.00543-26

**Published:** 2026-07-06

**Authors:** Jirui Zhang, Mengying Xu, Jiaojiao Mao, Jingjing Ma, Hui Wang

**Affiliations:** 1 Department of Oncology, The Fifth People’s Hospital of Suzhou/The Affiliated Infectious Diseases Hospital, Suzhou Medical College of Soochow University 74565, Suzhou, Jiangsu, China; 2 Department of Pharmacy, The Fourth Affiliated Hospital of Soochow University (Suzhou Dushu Lake Hospital) 606537, Suzhou, Jiangsu, China; University Children's Hospital Münster, Münster, Germany

**Keywords:** isavuconazole, invasive mold disease, hematological malignancies, real-world study, competing risk analysis

## Abstract

Isavuconazole is approved for invasive aspergillosis (IA) and mucormycosis, but real-world data in patients with hematological malignancies are limited. This retrospective study (2022–2025) included adult patients with hematological malignancies and proven/probable invasive mold disease (IMD) who were treated with isavuconazole for ≥72 h at two Chinese hospitals. The primary endpoint was week 6 treatment success (survival, clinical/radiological improvement, and no discontinuation due to failure/toxicity). Secondary endpoints included all-cause and IMD-attributable mortality at weeks 6 and 12 (competing risk analysis). Among 84 patients, 62 had aspergillosis (69.4% treatment success), 14 had mucormycosis (35.7%), and 8 had mixed infection (25.0%); the overall week 6 treatment success was 59.5% (50/84), with significant variation across infection types (*P* = 0.007) and treatment strategy (monotherapy 65.6% vs. combination 43.5%; *P* < 0.001), but not by therapy role. Week 6 all-cause and IMD-attributable mortality rates were 17.9% and 11.9%, respectively; IMD-attributable mortality rate was lower with monotherapy (6.6%) than with combination therapy (26.1%; *P* = 0.023). Week 12 all-cause mortality rate was 21.6%. Competing risk analysis showed significant differences in week 12 attributable mortality by infection type (overall *P* = 0.04): aspergillosis, 9.7%; mucormycosis, 36.0%; and mixed, 13.0%. Monotherapy was associated with lower attributable mortality than combination therapy (8.2% vs. 31.0%; *P* = 0.01). Four patients (4.8%) had drug-related adverse events (one discontinuation due to vomiting). In this real-world Chinese cohort, isavuconazole achieved 59.5% treatment success with favorable safety. Infection type and treatment strategy were key prognostic factors: aspergillosis and monotherapy were associated with better outcomes, whereas mucormycosis and combination therapy were associated with higher attributable mortality risk.

## INTRODUCTION

Invasive mold disease (IMD) remains a life-threatening complication in patients with hematological malignancies, particularly those with prolonged neutropenia or a history of allogeneic hematopoietic cell transplantation (HCT) (1, 2). Despite advances in antifungal prophylaxis and therapy, IMD is associated with substantial morbidity and mortality, with rates often exceeding 30%–50% in high-risk populations, making it a leading cause of infection-related death in this vulnerable group (3, 4).

The 2016 Infectious Diseases Society of America guideline recommends voriconazole as first-line therapy for invasive aspergillosis (IA), with alternative or salvage options including liposomal amphotericin B (LAmB), itraconazole, or caspofungin (5). More recent guidelines for patients with hematological malignancies, such as the 2019 guideline of the Infectious Diseases Working Party of the German Society for Hematology and Medical Oncology (AGIHO) (6) and the 2017 European Conference on Infections in Leukaemia (ECIL-6) guidelines (7), recommend either voriconazole or isavuconazole as first-line treatment for IA. For mucormycosis, the 2019 global guideline recommends isavuconazole as a suitable alternative to LAmB, especially for patients requiring prolonged therapy or those with dose-limiting nephrotoxicity (8). However, voriconazole has notable limitations: unpredictable pharmacokinetics requiring therapeutic drug monitoring, significant drug-drug interactions, and a high incidence of hepatotoxicity, visual disturbances, and neurological adverse events (5). Moreover, the emergence of azole-resistant *Aspergillus* strains and breakthrough infections despite prophylaxis with agents such as posaconazole have further complicated clinical management (9–12). Consequently, many patients experience first-line treatment failure or intolerance, underscoring an urgent need for effective and well-tolerated antifungal strategies.

Isavuconazole is a broad-spectrum triazole antifungal agent approved for the treatment of IA and mucormycosis (13). Compared with other azoles, isavuconazole offers several advantages, including a broader antifungal spectrum, more predictable pharmacokinetics, favorable oral bioavailability and tissue penetration, a lower incidence of adverse effects, and fewer drug-drug interactions (14). In the phase III SECURE trial, isavuconazole demonstrated non-inferior efficacy to voriconazole as first-line therapy, with a better tolerability profile regarding treatment-emergent adverse events and discontinuation rates (15).

Although randomized controlled trials (RCTs) provide pivotal evidence, their generalizability to real-world clinical practice remains limited (16). RCTs typically enroll highly selected patient populations and adhere to strict protocol-driven regimens, which may not capture the complexity of patients encountered in routine practice—particularly those requiring salvage therapy after prior antifungal treatment or those experiencing breakthrough infections while on prophylaxis. To date, several real-world studies have reported on isavuconazole use in patients with hematological malignancies (17–20), but data on its utilization patterns and clinical outcomes across different clinical scenarios (first-line therapy, salvage therapy, and management of breakthrough infections) and treatment strategies (monotherapy versus combination therapy) remain relatively limited, especially in the context of mixed infections and competing risk analysis.

Therefore, we conducted this two-center retrospective study to evaluate the real-world efficacy and safety of isavuconazole in patients with hematological malignancies and IMD. The specific objectives were to (i) characterize isavuconazole use across different clinical scenarios, including first-line therapy, salvage therapy, and treatment of breakthrough infections during antifungal prophylaxis; (ii) compare clinical outcomes between monotherapy and combination therapy; (iii) assess the impact of different infection types (aspergillosis, mucormycosis, and mixed infection) on treatment outcomes; (iv) quantify all-cause and IMD-attributable mortality using competing risk analysis to accurately estimate attributable risk; and (v) provide evidence-based insights to optimize antifungal strategies in complex real-world settings where data are limited.

## MATERIALS AND METHODS

### Study design and data collection

This two-center, retrospective, observational study included patients with hematological malignancies and IMD who received isavuconazole at two tertiary hospitals between January 2022 and December 2025. Patients were identified by searching the electronic medical record system for all medication orders of isavuconazole (both intravenous and oral formulations) during the study period. Both centers are Grade 3 Class A tertiary hospitals serving as regional referral centers, and their respiratory departments are provincial key clinical specialties that routinely manage pulmonary infections in patients with hematological malignancies. The study protocol was approved by the ethics committees of both participating institutions (The Fourth Affiliated Hospital of Soochow University, approval number 260047; The Fifth People’s Hospital of Suzhou, approval number ZF-2026-008-01). Owing to the retrospective nature of the study, the requirement for informed consent was waived.

Inclusion criteria were as follows: (i) age ≥18 years; (ii) proven or probable diagnosis of IMD according to the revised 2020 EORTC/MSG criteria (21); (iii) receipt of isavuconazole for at least 72 h, administered either intravenously or orally; and (iv) underlying diagnosis of hematological malignancy or receipt of HCT. All HCT recipients underwent transplantation for hematological malignancies. Exclusion criteria were isavuconazole used solely for prophylaxis, pregnancy or lactation, and incomplete clinical data.

Clinical data were extracted from the electronic medical record system, including demographic characteristics (age and sex), underlying hematological disease type and disease status (newly diagnosed/remission vs. relapsed/refractory), type of HCT (allogeneic vs. autologous) and time since HCT, presence of graft-versus-host disease (GVHD), and immunosuppressive regimens (corticosteroids converted to prednisone-equivalent dose, tacrolimus, cyclosporine, etc.) with their doses.

Infection-related characteristics included IMD type (aspergillosis, mucormycosis, or mixed infection), site of infection (pulmonary, rhino-orbital-cerebral, or disseminated), and microbiological evidence (culture, histopathology, serum, or bronchoalveolar lavage fluid [BALF] galactomannan [GM] assay [Platelia Aspergillus enzyme immunoassay [Bio-Rad]; positivity: serum index ≥0.5, BALF index ≥1.0], metagenomic next-generation sequencing results). Chest computed tomography (CT) findings (nodules, halo sign, cavitation, airway involvement, and pleural effusion) were also recorded.

Regarding antifungal therapy, the isavuconazole regimen (loading and maintenance doses), start and stop dates, route of administration (oral or intravenous), and treatment duration were collected. Treatment indication was categorized as first-line therapy (including empiric/targeted therapy and treatment of breakthrough infection during antifungal prophylaxis), salvage therapy (switched from prior antifungal therapy due to toxicity or lack of efficacy), or oral step-down therapy. Combination therapy was defined as overlapping use of isavuconazole with another systemic antifungal agent for ≥72 h.

Laboratory parameters included absolute neutrophil count (ANC). Neutropenia was defined as ANC < 0.5 × 10⁹/L, and prolonged neutropenia as duration >10 days. Liver function (alanine aminotransferase [ALT], aspartate aminotransferase [AST], and total bilirubin [TB]) and renal function (creatinine and estimated glomerular filtration rate) were assessed at baseline, day 7, day 14, month 1, and month 3 of therapy. For safety assessment, the type and management (discontinuation, dose reduction, or symptomatic treatment) of treatment-emergent adverse events were recorded.

For efficacy assessment, the primary endpoint was the overall treatment success rate at week 6, defined as a composite endpoint comprising survival, clinical cure/improvement, and absence of treatment discontinuation due to failure or toxicity. Secondary endpoints included all-cause mortality and IMD-attributable mortality at weeks 6 and 12. Follow-up continued until week 12 after treatment initiation, death, or loss to follow up; patients lost to follow up were censored at their last known alive date.

### Study definitions

The diagnosis of invasive fungal disease was established according to the criteria of the European Organization for Research and Treatment of Cancer and the Mycoses Study Group Education and Research Consortium (EORTC/MSGERC) (21). Clinical efficacy was assessed at week 6 after initiation of isavuconazole therapy, based on clinical symptoms, laboratory findings, and radiological imaging. Treatment outcomes were categorized as success or failure. Treatment success included complete response (CR) and partial response (PR). CR was defined as complete resolution of clinical symptoms and signs, with radiologic findings showing only stable residual scarring or non-progressive fibrotic changes, as complete radiological normalization is often not attained within 6 weeks after antifungal therapy. PR was defined as marked clinical improvement with significant resolution of symptoms and laboratory abnormalities, accompanied by a ≥ 50% improvement in radiological findings, although some residual abnormalities might persist. Treatment failure was defined as lack of significant clinical or radiological improvement, disease progression, or death. The overall treatment response rate was calculated as (number of patients with CR + number with PR)/total number of patients × 100%.

Safety evaluation focused on any adverse events that were not present before isavuconazole therapy or any pre-existing events that worsened in intensity or frequency following treatment initiation. Nephrotoxicity was defined as an increase in serum creatinine level of at least 0.55 mg/dL or a ≥ 50% increase from baseline (22). Hepatotoxicity was diagnosed when there was simultaneous elevation of AST, alkaline phosphatase, and TB levels, with at least one exceeding twice the upper limit of normal (ULN), or when ALT or conjugated bilirubin levels exceeded twice the ULN after exclusion of viral hepatitis and other causes of liver injury (23). Other adverse events, including nausea, vomiting, rash, diarrhea, visual disturbances, and hypokalemia, were identified based on the drug prescribing information and descriptions recorded in the medical records.

For subgroup analyses, infection type was defined as aspergillosis, mucormycosis, or mixed infection (both *Aspergillus* and Mucorales); treatment strategy as monotherapy (isavuconazole alone) versus combination therapy (isavuconazole with one or two additional antifungal agents, including nebulized or systemic formulations, for ≥72 h); and therapy role as first-line (initial therapy, including breakthrough infection) versus other indications (salvage therapy for prior antifungal toxicity or refractoriness, or step-down from other intravenous antifungals to oral isavuconazole).

### Statistical analysis

For continuous variables, normality was assessed prior to analysis; variables following a normal distribution were expressed as mean ± standard deviation (SD), whereas those with a non-normal distribution were presented as median with interquartile range [M (Q1, Q3)]. Categorical variables were summarized as frequencies (percentages). For comparisons between groups, normally distributed continuous variables with homogeneity of variance were analyzed using the Student’s *t*-test; otherwise, the non-parametric Mann-Whitney U test was applied. Categorical variables were compared using the χ² test or Fisher’s exact test, as appropriate. The primary endpoint, week-6 treatment success rate, was reported as a percentage with its 95% CI. Subgroup analyses were performed according to infection type, therapy role, and treatment strategy, with intergroup comparisons conducted using the χ² test. For survival analysis, week-12 all-cause mortality was estimated using the Kaplan-Meier method, and survival curves were plotted; comparisons between groups were performed using the log-rank test, and patients lost to follow up were censored at their last known alive date. Given that non-IMD death represents a competing event, week-12 IMD-attributable mortality was estimated using a competing risk model to calculate the cumulative incidence function , with intergroup comparisons performed using Gray’s test; the results were presented as cumulative incidences with 95% CIs. The incidence of adverse events was described as frequencies (percentages). All statistical analyses were performed using SPSS version 26.0 (IBM Corp., Armonk, NY, USA) and R software version 4.2.1 (R Foundation for Statistical Computing, Vienna, Austria). A two-sided *P*-value < 0.05 was considered to indicate statistical significance.

## RESULTS

### Clinical characteristics of the study population

**Fig 1:**
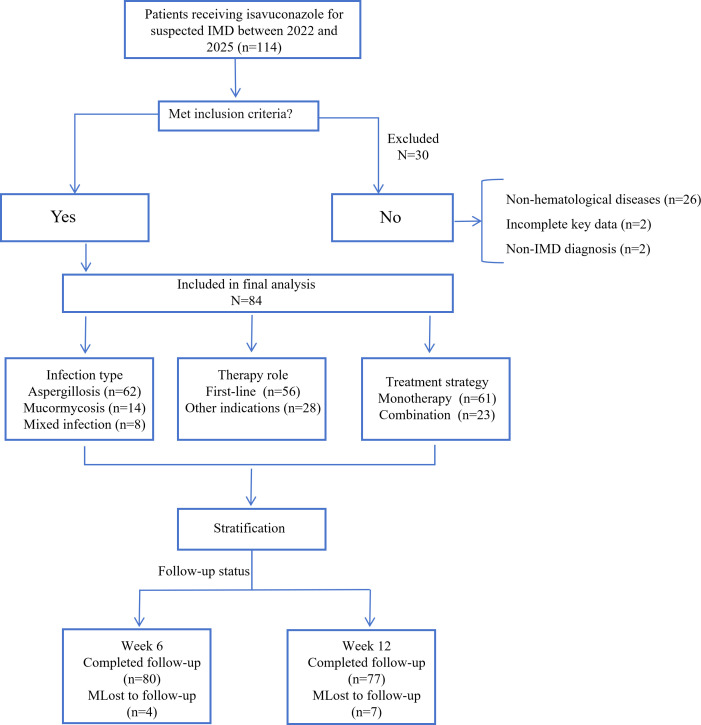
Patient selection flow diagram. A total of 114 patients who received isavuconazole for suspected invasive mold disease (IMD) between 2022 and 2025 were screened. After exclusion of 30 patients (26 with non-hematological diseases, 2 with incomplete key data, and 2 with non-IMD diagnoses), 84 patients were included in the final analysis. IMD, invasive mold disease.

A total of 114 patients who received isavuconazole for suspected IMD between 2022 and 2025 were screened. After excluding 30 patients (26 with non-hematological diseases, 2 with incomplete data, and 2 with non-IMD diagnoses), 84 patients were included in the final analysis (Fig. 1). The baseline characteristics of these 84 patients are summarized in Table 1.

**TABLE 1:** Baseline demographic and clinical characteristics of the study cohort (*N* = 84)^*c*^

Variable	
Age, mean ± SD, years	45.7 ± 16.7
Female gender, n (%)	39 (46.4%）
Type of hematological malignancy, n (%)	
Acute myeloid leukemia (AML)	45 (53.6%）
acute lymphoblastic leukemia (ALL)	8 (9.5%）
myelodysplastic neoplasia (MDS)	9 (10.7%）
aplastic anemia (AA）	4 (4.7%）
Others^*a*^	18 (21.4%）
Disease status at IMD onset, n (%)	
Relapsed/refractory	18 (21.4%）
Non-relapsed (newly diagnosed/remission)	66 (78.6%）
HCT, n(%)	45 (53.6%）
The type of HCT, n(%)	
alloHCT	40 (88.9%）
Autologous HCT	5 (11.1%）
GVHD, n(%)	32 (38.1%）
Immunosuppressive regimen at diagnosis of IMD, n (%)	
Corticosteroids	23 (27.4%）
Daily prednisone dose or equivalent, median(IQR), mg	5 (5-10)
Tacrolimus	12 (14.3%）
Daily dose, mg/day, median (IQR)	1.0 (0.5-2)
Cyclosporine	2 (2.4%）
Daily dose, mg/day, median (range)	42.5 (30.0-55.0)
Major comorbidities, n (%)	
Diabetes mellitus	14 (16.7%）
Chronic renal disease	8 (9.5%）
Chronic heart disease	4 (4.8%）
Chronic liver disease	8 (9.5%）
Neutropenia status at IMD onset	
Neutropenia, n (%)	27 (32.1%）
Duration of neutropenia, median (IQR), d^*b*^	7 (3-21）
Prolonged neutropenia (>10 days), n (%)	8 (9.5%）
Other post-transplant complications, n(%)	
CMV infection or disease	13 (15.5%）
Time interval to IMD diagnosis, median (IQR), d	8 (5–41)
COVID-19	3 (3.6%）

^*a*^ Others included: lymphoma (*n* = 10), chronic myeloid leukemia (*n* = 4), multiple myeloma (*n* = 2), and primary myelofibrosis (*n* = 2).

^*b*^ Duration of neutropenia was calculated among patients with neutropenia (*n* = 27), defined as the time from the first documented ANC < 0.5 × 10⁹/L to the date of IMD diagnosis.

^*c*^ ANC, absolute neutrophil count; IMD, invasive mold disease; HCT, hematopoietic cell transplantation; GVHD, graft-versus-host disease.

The mean age at IMD diagnosis was 45.7 ± 16.7 years. Acute myeloid leukemia (AML) was the most common underlying hematological malignancy, accounting for 45 patients (53.6%), followed by myelodysplastic neoplasia (MDS) in 9 (10.7%) and acute lymphoblastic leukemia (ALL) in 8 (9.5%). HCT had been performed in 45 patients (53.6%), of whom 40 (88.9%) underwent allogeneic HCT. GVHD occurred in 32 patients (38.1%). Immunosuppressive regimens included corticosteroids in 23 patients (27.4%), tacrolimus in 23 (27.4%), and cyclosporine in 2 (2.4%); 13 patients (15.5%) received dual or triple immunosuppressive therapy.

At the time of IMD diagnosis, 27 patients (32.1%) had neutropenia. Among these patients, the median duration of neutropenia was 7 days (interquartile range [IQR], 3–21 days). Prolonged neutropenia (>10 days) was observed in 8 patients (9.5% of the total cohort).

Regarding comorbidities, diabetes mellitus was present in 14 patients (16.7%), chronic kidney disease in 8 (9.5%), chronic liver disease in 8 (9.5%), and chronic heart disease in 4 (4.8%). Among post-transplant complications, cytomegalovirus (CMV) infection occurred in 13 patients (15.5%), with a median interval of 8 days (IQR, 5–41 days) from CMV infection to IMD diagnosis. Concurrent COVID-19 infection at the time of IMD diagnosis was documented in 3 patients (3.6%).

### Characteristics of IMD

The clinical, radiological, and microbiological features of IMD are summarized in Table 2. Among the 84 patients, 62 (73.8%) had IA, 14 (16.7%) had mucormycosis, and 8 (9.5%) had mixed infection. Chest CT was performed in all patients. The radiological features observed in mucormycosis and mixed infection were qualitatively similar to those reported for aspergillosis (e.g., nodules and cavitation), with no extrapulmonary involvement documented. The median time from HCT to IMD diagnosis was 730 days (IQR, 363–1,095 days) for IA, 540 days (IQR, 240–730 days) for mucormycosis, and 550 days (range, 370–730 days) for mixed infection (*n* = 2).

**TABLE 2:** Clinical, radiological, and microbiological characteristics of IMD episodes in patients with hematological malignancies^*b*^

Variable	
IA	*n* = 62
Time interval from HCT, median (IQR), d	730 (362.75-1095）
Certainty of diagnosis according to EORTC/MSG, n (%)	
Probable	55
Proven	7
Proven cases by diagnostic method	
Histopathology	4
Sterile-site culture (pleural fluid)	3
Findings on thoracic CT scan, n (%) **^*a*^**	
Dense nodules	32/62 (51.6%)
>5 nodules	12/62 (19.4%)
Cavitation	10/62 (16.1%)
Airway involvement	37/62 (59.7%)
Halo sign	11/62 (17.7%）
Pleural effusion	22/62 (35.5%）
Bilateral pulmonary involvement	50/62 (80.6%）
Diagnostic procedures, n (%)	
Detection of serum GM	61
Serum level, median (IQR), ODs	0.17 (0.1-0.51）
Positive (index ≥0.5 ODs)	15/61 (24.6%）
Detection of GM in BALF	34
BALF level, median (IQR), ODs	1.23 (0.385-4.21）
Positive (index ≥1.0 ODs)	18/34 (52.9%）
Bronchial aspirate culture	58
Positive identification	26/58 (44.8%）
BALF culture	28
Positive identification	8/28 (28.6%）
Tissue biopsy culture	5
Positive identification	4
*Aspergillus* mNGS assay	43
Positive identification	38/43 (88.4%）
Isolated species, n (%)	
*A. fumigatus.*	43
*A. flavus.*	11
*A. niger.*	5
Other	3
Mucormycosis	*n* = 14
Time interval from HCT, median (IQR)	540 (240-730）
Certainty of diagnosis according to EORTC/MSG, n (%)	
Probable	12
Proven	2
Proven cases by diagnostic method	
Histopathology	2
Diagnostic procedures, n (%)	
Tissue biopsy culture	2
Positive identification	2
*Aspergillus* mNGS assay	14
mNGS positive, n (%)	14 (100%）
*Rhizopus* spp.	2
*Lichtheimia* spp.	5
*Rhizomucor* spp.	5
*Cunninghamella* spp.	2
Mixed infection (*Aspergillus* and Mucorales)	*n* = 8
Time interval from HCT, median (range)	550 (370-730）
Certainty of diagnosis according to EORTC/MSG, n (%)	
Probable	8 (100%）
Diagnostic procedures, n (%)	
mNGS positive, n (%)	8 (100%）
Isolated species, n (%)	
*Rhizomucor* spp.	5
*Rhizopus* spp.	1
*Lichtheimia* spp.	2
*A. fumigatus.*	5
*A. flavus.*	2
*A. niger.*	1

^*a*^ Chest CT was performed in all patients. Findings for mucormycosis and mixed infection are described in the Results section (3.2).

^*b*^ IA, invasive aspergillosis; GM, galactomannan; BALF, bronchoalveolar lavage fluid; mNGS, metagenomic next-generation sequencing.

According to the EORTC/MSG criteria, the majority of IA cases were classified as probable (88.7% [55/62]), with the remaining seven (11.3%) classified as proven. Of these seven proven cases, four were confirmed by histopathology (lung biopsy) and three by positive culture from a sterile site (pleural fluid). The most frequently isolated *Aspergillus* species was *A. fumigatus*, accounting for 69.4% (43/62) of IA cases, followed by *A. flavus* (17.7% [11/62]) and *A. niger* (8.1% [5/62]). Among IA patients, serum GM was detected in 61 patients (98.4%), with a median index of 0.17 (IQR: 0.1-0.51); positivity (index ≥0.5) was observed in 15 patients (24.6%). BALF GM was tested in 34 patients, with a median index of 1.23 (IQR: 0.385–4.21) and a positivity rate (index ≥1.0) of 52.9% (18/34). Metagenomic next-generation sequencing (mNGS) was performed in 43 IA patients, yielding positive identification in 38 (88.4%).

For mucormycosis, the majority of cases were classified as probable (85.7% [12/14]), with 2 (14.3%) classified as proven. mNGS was performed in all 14 patients (100%), all of whom tested positive. The most commonly identified Mucorales species were *Lichtheimia spp. and Rhizomucor spp.,* each accounting for 35.7% (5/14), followed by *Rhizopus spp.* (14.3% [2/14]) and *Cunninghamella spp.* (14.3% [2/14]).

In the mixed infection group (*n* = 8), all patients (100%) were classified as probable according to the EORTC/MSG criteria, as none underwent tissue biopsy for histopathologic confirmation. mNGS identified both *Aspergillus* and Mucorales in all 8 patients (100%). The most frequently detected species were *A. fumigatus* (62.5% [5/8]), *Rhizomucor spp*. (62.5% [5/8]), *Lichtheimia spp*. (25.0% [2/8]), and *A. flavus* (25.0% [2/8]).

A detailed patient-level listing of microbiological tests and positive results is provided in Table S1.

### Description of isavuconazole therapy course

The treatment characteristics and safety outcomes are summarized in Table 3. The mean duration of isavuconazole therapy was 57.1 ± 94.2 days. Regarding treatment duration, 4 patients (4.8%) received therapy for less than 7 days, 29 (34.5%) for 7–14 days, 22 (26.2%) for >14–28 days, 9 (10.7%) for >28–56 days, and 20 (23.8%) for more than 56 days.

**TABLE 3:** Details on the course of isavuconazole therapy and safety outcomes

Variable	
Length of therapy, mean ± SD, d	57.13 ± 94.21
Time periods of therapy, n (%)	
<7 d	4 (4.8%）
7–14 d	29 (34.5%）
14–28 d	22 (26.2%）
28–56 d	9 (10.7%）
>56 d	20 (23.8%）
Indication, n (%)	
First-line therapy	56 (66.7%）
Empiric/targeted therapy (no recent azole)	45/56 (80.4%)
Breakthrough infection on antifungal prophylaxis	11/56 (19.6%)
Salvage therapy due to treatment-emergent toxicity of previous antifungal agent(s)	7 (8.3%）
Salvage therapy due to refractory disease to previous antifungal agent(s)	12 (14.3%）
Oral step-down therapy	9 (10.7%）
Initial mode of administration, n (%)	
Oral	57 (67.9%）
Intravenous	27 (32.1%）
Type of antifungal regimen, n (%)	
Monotherapy	61 (72.6%）
Combination therapy	23 (27.4%）
Isavuconazole + Intravenous ABCD	8/23 (34.8%）
Isavuconazole + Nebulized L-AmB	5/23 (21.7%）
Isavuconazole +caspofungin	9/23 (39.1%）
Isavuconazole + caspofungin + intravenous ABCD	1/23 (4.3%）
Occurrence of ≥1 TEAE, n (%)	4 (4.8%)
Liver enzyme elevation	2 (2.4%）
Vomiting	1 (1.2%）
Phlebitis	1 (1.2%）
TEAE requiring premature discontinuation of isavuconazole therapy, n (%)	1 (1.2%）

Isavuconazole was administered as first-line therapy in 56 patients (66.7%), of whom 45 (80.4%) received empiric or targeted therapy without recent azole exposure, and 11 (19.6%) were treated for breakthrough infection during antifungal prophylaxis. Salvage therapy was administered in 19 patients (22.6%), including 7 (8.3%) due to treatment-emergent toxicity of prior antifungal agents and 12 (14.3%) due to refractory disease to previous antifungal therapy. Oral step-down therapy was used in 9 patients (10.7%). The initial route of administration was oral in 57 patients (67.9%) and intravenous in 27 (32.1%).

Regarding treatment strategy, monotherapy was used in 61 patients (72.6%), while combination therapy was employed in 23 (27.4%). Among those receiving combination therapy, the most common partners were caspofungin (39.1% [9/23]), intravenous amphotericin B cholesterol sulfate complex (ABCD) (34.8% [8/23]), and nebulized amphotericin B deoxycholate (AmB-D) (21.7% [5/23]). A triple regimen consisting of isavuconazole, caspofungin, and intravenous ABCD was administered in one patient (4.3%).

Safety outcomes are also presented in Table 3. Treatment-emergent adverse events (TEAE) occurred in 4 patients (4.8%), including liver enzyme elevation (*n* = 1), vomiting (*n* = 1), and phlebitis (*n* = 1). One patient (1.2%) required premature discontinuation of isavuconazole therapy due to intolerable vomiting.

### Efffcacy of isavuconazole therapy

#### Primary efficacy endpoint: week 6 treatment success

**Fig 2:**
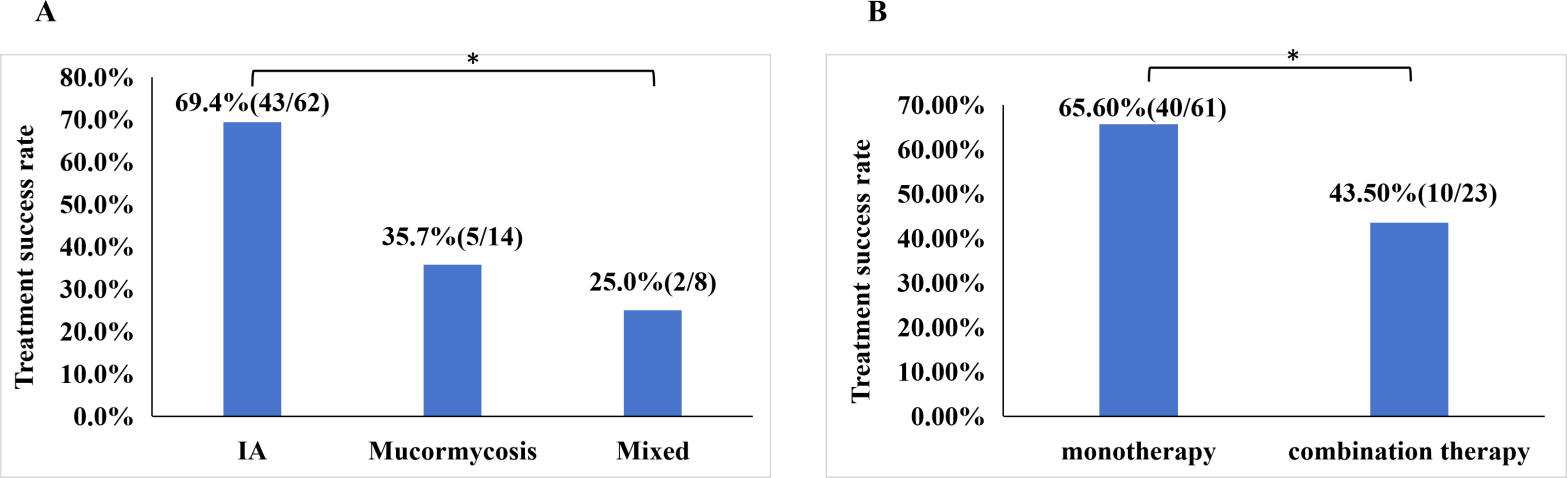
Treatment success rates at week 6 after initiation of isavuconazole therapy. (**A**) According to infection type (IA, mucormycosis, and mixed infection. (**B**) According to treatment strategy (monotherapy, combination therapy). Data are presented as percentages. Numbers above bars indicate the number of patients achieving treatment success over the total number in each subgroup.*P-value < 0.05. IA, invasive aspergillosis.

The primary endpoint, week 6 treatment success rate was 59.5% (50/84) (Table 4). As shown in Fig. 2, treatment success varied significantly by infection type (overall *P* = 0.007): the IA group had the highest success rate (69.4% [43/62]), followed by mucormycosis (35.7% [5/14]) and mixed infection (25.0% [2/8]). The success rate was significantly higher in the monotherapy group than in the combination therapy group (65.6% [40/61] vs. 43.5% [10/23]; *P* < 0.001). No significant difference was observed between first-line and other indications groups (60.7% [34/56] vs. 57.1% [16/28]; *P* = 0.467).

**TABLE 4:** Primary and secondary efficacy outcomes^*a*^

Outcome measure	Overall (*N* = 84)	By therapy role		By infection type			By treatment strategy	
		First-line therapy (*n* = 56)	other indication (*n* = 28)	IA (*n* = 62)	Mucormycosis (*n* = 14)	Mixed (*n* = 8)	Monotherapy (61)	Combination therapy (23)
Primary endpoint								
Week 6 treatment success, n (%)	59.5% (50/84）	60.7% (34/56）	57.1% (16/28）	69.4% (43/62）	35.7% (5/14）	25.0% (2/8）	65.6% (40/61）	43.5% (10/23）
*P-*value		0.467		0.007			＜0.001	
Secondary endpoints								
Week 6 all-cause mortality, n (%)	17.9% (15/84）	19.6% (11/56）	14.3% (4/28）	14.5% (9/62）	28.6% (4/14）	25% (2/8）	13.1% (8/61）	30.4% (7/23）
Week 6 IMD-associated mortality, n (%)	11.9% (10/84）	10.7% (6/56）	14.3% (4/28）	8.1% (5/62）	28.6% (4/14）	12.5% (1/8）	6.6% (4/61）	26.1% (6/23）
*P-*value (IMD-associated*)*		0.441		0.084			0.023	
Week 12 all-cause mortality, % (95% CI)^*b*^	21.6% (12.78%–30.42%）	23.55 (12.33%–34.67%)	17.9% (3.79%–32.01%)	16.1% (6.9%–25.3%)	42.9% (17%–68.8%）	25% (0%–54.9%）	16.40 (7.19%–25.61%）	35.10 (15.5%–54.70%)
*P-*value (log-rank)		0.568		0.087			0.069	
Week 12 IMD-associated mortality, % (95% CI)^*c*^	14% (7.8%, 23%）	13% (5.5%, 23%）	18% (6.3%, 34%）	9.7% (3.9%, 19%）	36% (12%, 60%）	13% (0.48%, 44%）	8.2% (3.0%, 17%）	31% (13%, 50%）
*P*-value (Gray’s test)		0.5		0.04			0.01	

^*a*^ *P* values were calculated using Pearson’s χ test or Fisher’s exact test as appropriate based on expected cell counts. Overall *P* for infection type comparison.

^*b*^ Estimated by Kaplan-Meier method.

^*c*^ Estimated by cumulative incidence function for competing risks.

#### Secondary efficacy endpoints: mortality outcomes

**Fig 3:**
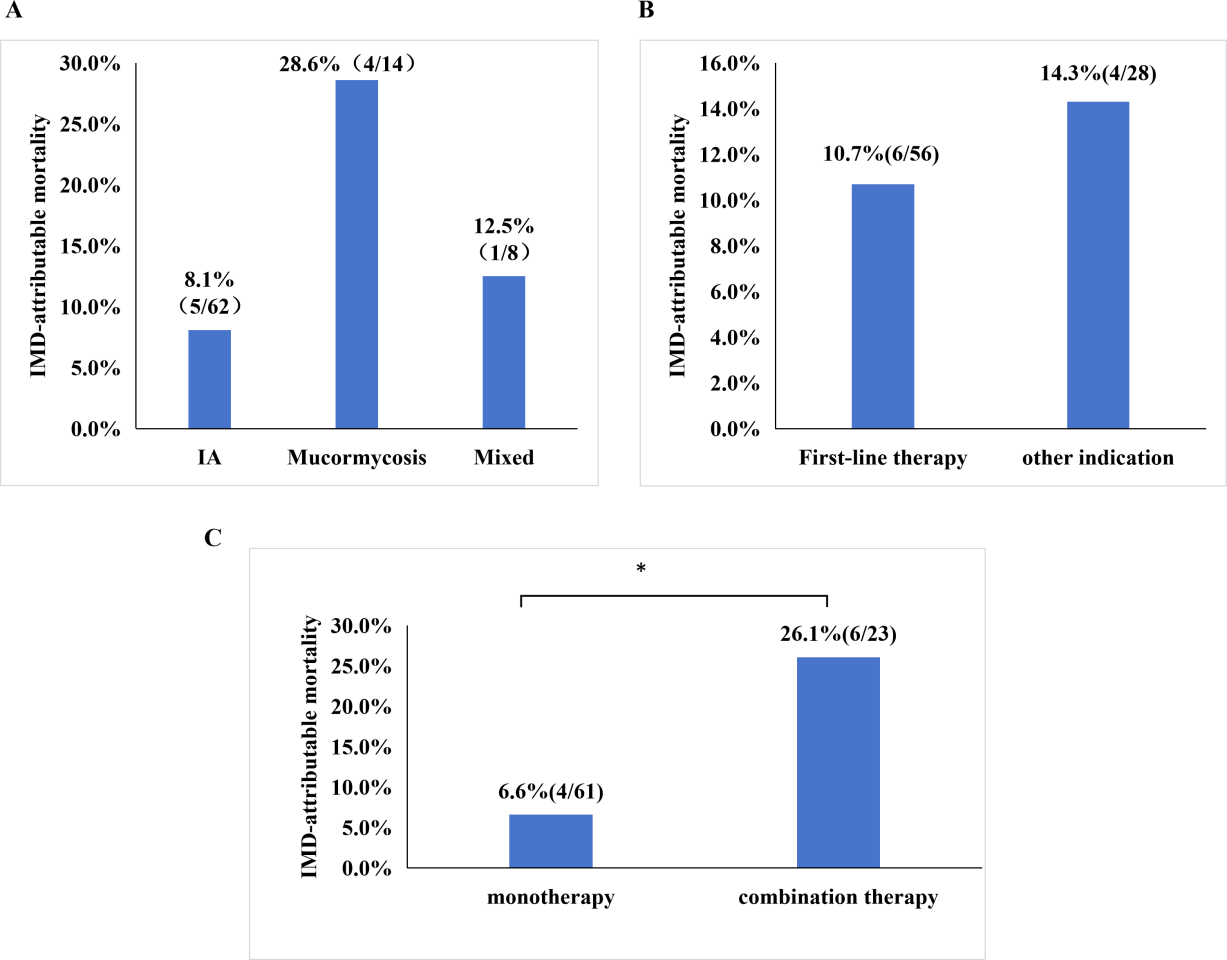
IMD-associated mortality at week 6 after initiation of isavuconazole therapy. (**A**) According to infection type (IA, mucormycosis, and mixed infection). (**B**) According to therapy role (first-line therapy vs. other indications). (**C**) According to treatment strategy (monotherapy vs. combination therapy). Data are presented as percentages. Numbers above bars indicate the number of patients with IMD-associated death over the total number in each subgroup.*P < 0.05. IA, invasive aspergillosis; IMD, invasive mold disease.

At week 6, all-cause mortality was 17.9% (15/84), and IMD-associated mortality was 11.9% (10/84) (Table 4). As shown in Fig. 3A, the IMD-associated mortality rate varied by infection type, with the highest rate observed in the mucormycosis group (28.6% [4/14]), followed by mixed infection (12.5% [1/8]) and IA (8.1% [5/62]); however, the overall difference did not reach statistical significance (*P* = 0.084). No significant difference was observed between first-line therapy and other indication groups (10.7% [6/56] vs. 14.3% [4/28]; *P* = 0.441) (Fig. 3B). Notably, as shown in Fig. 3C, the IMD-associated mortality rate was significantly higher in the combination therapy group than in the monotherapy group (26.1% [6/23] vs. 6.6% [4/61]; *P* = 0.023).

**Fig 4:**
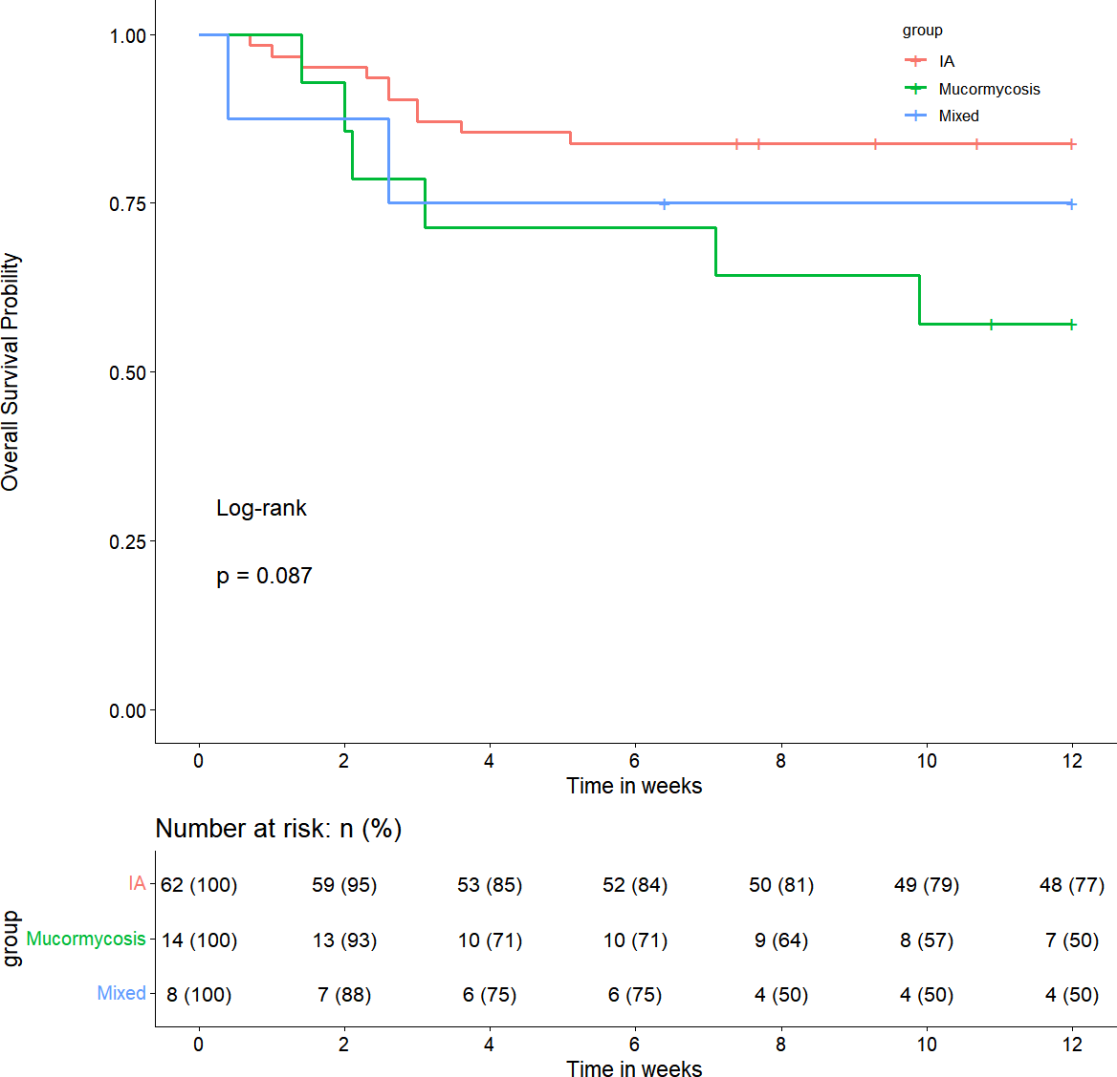
Kaplan-Meier curves for week 12 all-cause mortality stratified by infection type. Kaplan-Meier estimates of cumulative all-cause mortality are shown for patients with IA (*n* = 62), mucormycosis (*n* = 14), and mixed infection (*n* = 8). The risk table displays the number of patients at risk at weeks 0, 4, 8, and 12. Tick marks indicate censored patients. IA, invasive aspergillosis. Pairwise log-rank *P*-values are provided in Table S2.

Kaplan-Meier analysis estimated the week 12 all-cause mortality at 21.6% (95% CI: 12.78%–30.42%) overall. As shown in Fig. 4, the mucormycosis group had the highest mortality rate (42.9%), followed by mixed infection (25.0%) and IA (16.1%), although the difference did not reach statistical significance (log-rank *P* = 0.087). No significant differences in all-cause mortality were observed by therapy role (log-rank *P* = 0.568) or treatment strategy (log-rank *P* = 0.069) (Fig. S1). Pairwise log-rank comparisons are provided in Table S2.

**Fig 5:**
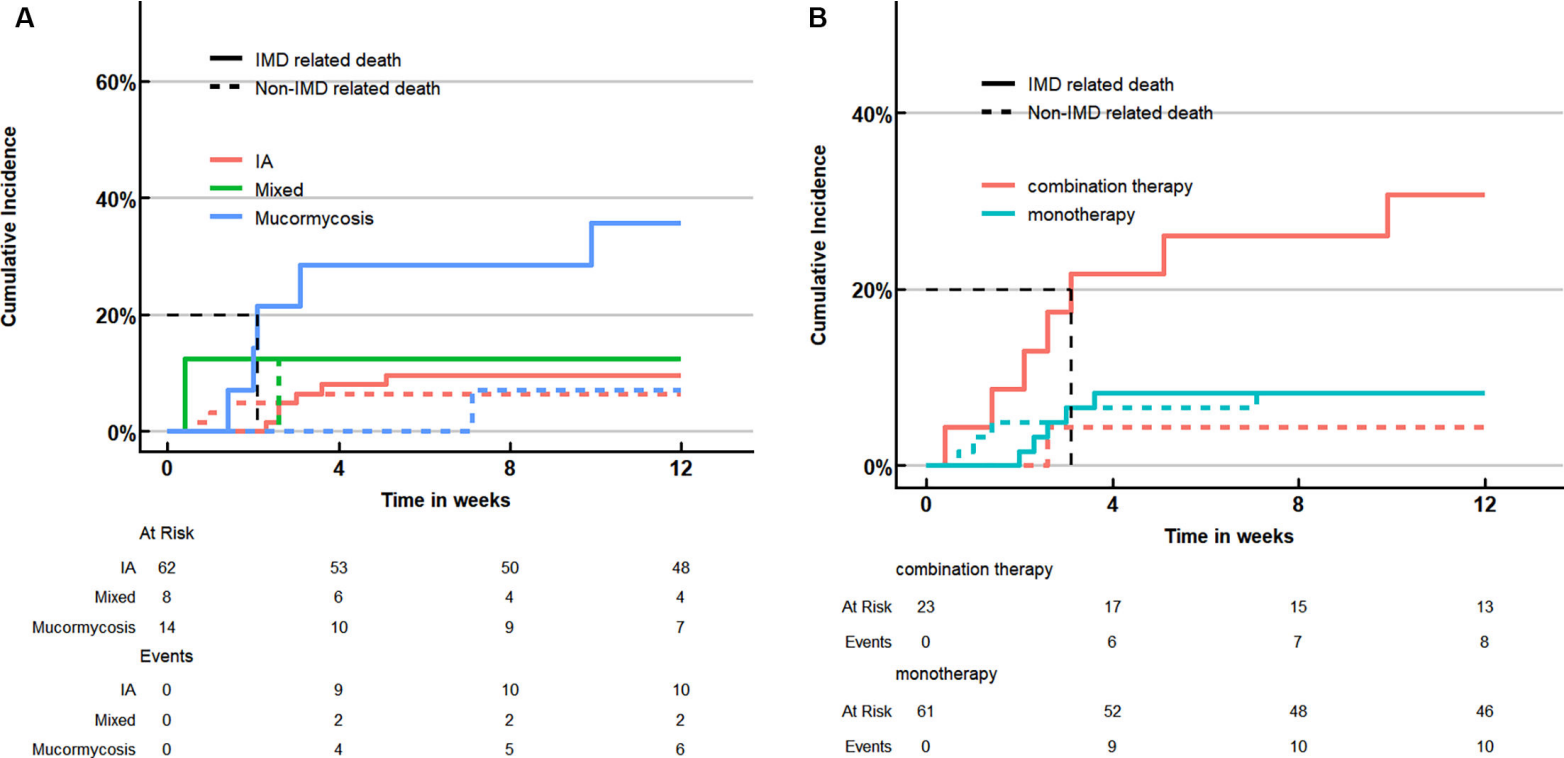
Cumulative incidence of IMD-associated mortality at week 12. (**A**) According to infection type (IA, mucormycosis, and mixed infection). (**B**) According to treatment strategy (monotherapy vs. combination therapy). Solid lines represent the cumulative incidence of IMD-associated mortality, and dashed lines represent non-IMD mortality as competing events. The risk tables display the number of patients at risk at weeks 0, 4, 8, and 12. Gray’s test *P*-values: (**A**) *P* = 0.04 for overall comparison among the three infection types. (**B**) *P* = 0.01 for monotherapy vs. combination therapy. IA, invasive aspergillosis; IMD, invasive mold disease.

Competing risk analysis was performed to estimate the cumulative incidence of IMD-associated mortality, accounting for non-IMD death as a competing event. As shown in Fig. 5A, the week 12 IMD-associated mortality varied significantly by infection type (Gray’s test *P* = 0.04). The mucormycosis group had the highest cumulative incidence (36.0% [95% CI: 12.0%–60.0%]), followed by mixed infection (13.0% [95% CI: 0.5%–44.0%]) and IA (9.7% [95% CI: 3.9%–19.0%]). Similarly, as shown in Fig. 5B, the IMD-associated mortality was significantly higher in the combination therapy group than in the monotherapy group (31.0% [95% CI: 13.0%–50.0%] vs. 8.2% [95% CI: 3.0–17.0%]; Gray’s test *P* = 0.01).

## DISCUSSION

To the best of our knowledge, this study represents one of the largest Chinese two-center cohorts to date evaluating the real-world efficacy and safety of isavuconazole for the treatment of IMD in patients with hematologic malignancies. Our findings demonstrated a week 6 treatment success rate of 59.5% (50/84), with favorable tolerability and a low incidence of treatment-emergent adverse events (4.8%). Notably, treatment outcomes varied significantly by infection type and treatment strategy: patients with IA and those receiving monotherapy exhibited better responses, whereas mucormycosis and combination therapy were associated with higher mortality risks.

### Comparison with previous real-world studies

The week 6 treatment success rate of 59.5% in our cohort was broadly consistent with findings from other real-world studies. Tuohuti et al. reported a week 6 favorable response rate of 57.6% in 66 Chinese patients with hematological malignancies and invasive fungal disease (24), which is highly concordant with our results. Luo et al. reported a clinical improvement rate of 64.0% in 25 Chinese patients with hematological malignancies (18), approximating the response rate observed in our IA subgroup (69.4%). Gandolpho et al. from Brazil reported a week 6 overall survival rate of 60% in 50 patients with hematological malignancies and IA (19), further supporting our findings.

Two recent real-world studies evaluated isavuconazole in Argentine and Brazilian cohorts (19, 20). Compared with these reports, our study has several distinguishing features. First, we exclusively included proven or probable IMD cases (100%), whereas the Argentine study included 59.5% possible IFI cases, which may have influenced response rates. Second, our cohort uniquely characterizes mixed *Aspergillus* and Mucorales infections (*n* = 8, 9.5%), a subgroup not addressed in previous real-world studies. Third, we applied competing risk analysis to estimate IMD-attributable mortality—a methodological refinement over conventional Kaplan-Meier estimation. The numerically lower treatment success rate in our cohort (59.5%) compared with the Argentine study (70.9%–91.4%) is likely attributable to the stricter diagnostic criteria applied in our study.

### Monotherapy versus combination therapy

Current evidence regarding monotherapy versus combination therapy for IMD remains limited. The 2025 American Thoracic Society guidelines, based on low-quality evidence, conditionally recommend either initial triazole monotherapy or initial triazole plus echinocandin combination therapy for invasive pulmonary aspergillosis (25). With respect to mucormycosis, a retrospective study using propensity score analysis failed to demonstrate clinical benefit from combination therapy (26). Among solid organ transplant recipients, the SOTIS study similarly showed superior outcomes with first-line isavuconazole monotherapy (27). These findings align with our observation of poorer outcomes in the combination therapy group (treatment success 43.5% vs. 65.6%, IMD-attributable mortality 26.1% vs. 6.6%). Notably, combination therapy was more frequently used in patients with relapsed/refractory disease (12 salvage cases in our cohort), reflecting clinical practice of intensifying treatment in more severe or refractory cases. Thus, the association between combination therapy and adverse outcomes likely reflects confounding by indication (i.e., more severe baseline illness) rather than a causal effect of the combination regimen itself. Nevertheless, the combination therapy group was heterogeneous with respect to the specific antifungal partner, and the small sample size precluded meaningful subgroup analysis. Therefore, our comparison of monotherapy versus combination therapy should be interpreted as an exploratory finding.

### Mucormycosis and mixed infections

Mucormycosis is a devastating invasive fungal infection with mortality rates ranging from 40% to 80%. According to the 2019 global guideline for the diagnosis and management of mucormycosis (8), isavuconazole is recommended with moderate strength as an alternative first-line treatment to liposomal amphotericin B, particularly for patients requiring prolonged therapy or those with dose-limiting nephrotoxicity. In our study, the week 6 treatment success rate in the mucormycosis group was 35.7% (5/14), and the week 12 IMD-attributable mortality was 36.0%. Compared with historical data, untreated mucormycosis has a mortality rate of nearly 100% (28), and a 2025 systematic review reported 42-day all-cause mortality of 33% in the VITAL trial and 24.6% for isavuconazole-only treatment across studies (29); these results reflect the real-world clinical value of isavuconazole.

Mixed *Aspergillus* and Mucorales infections (*n* = 8, 9.5%) represent a particularly challenging subgroup. Their week 6 treatment success rate was 25.0% (2/8), and the week 12 IMD-attributable mortality was 13.0% (95% CI 0.5%–44.0%). However, due to the very small sample size, these estimates are highly unstable; the wide confidence intervals overlap with those of mucormycosis alone (36.0%; 95% CI: 12.0%–60.0%), and no reliable comparison of survival probability can be made. The lower treatment success rate at week 6 suggests a poorer early response, but the attributable mortality was not higher than that of mucormycosis. Although the small sample size precludes definitive conclusions, these observations should be considered hypothesis-generating.

Of note, all IMD episodes in our cohort were pulmonary; no sinonasal, cutaneous, or disseminated IMD was documented. This may be explained by the respiratory specialty focus of the participating centers (both are tertiary hospitals with provincial key respiratory departments) and the fact that pulmonary involvement is the predominant presentation of IMD in patients with hematological malignancies, especially aspergillosis. However, we cannot exclude the possibility that isolated extrapulmonary IMD occurred but was not captured due to the absence of dedicated imaging for extrapulmonary sites or because such patients were managed by other specialties. This should be considered a limitation when interpreting our findings.

### Late-onset IMD after HCT

Regarding late-onset IMD after HCT, it should be noted that 88.9% of the patients who underwent transplantation received allogeneic HCT, many of whom had chronic graft-versus-host disease requiring prolonged immunosuppressive therapy. This explains why IMD could occur several years after HCT even when the underlying malignancy was in remission. In contrast, patients who received autologous HCT do not receive long-term immunosuppression for GVHD; in our small cohort (*n* = 5), all IMD events occurred within 12 months post-transplant.

### Competing risk analysis

The use of competing risk analysis to estimate IMD-attributable mortality represents a methodological strength of our study. Conventional Kaplan-Meier analysis treats non-IMD deaths as censored observations, which overestimates the cumulative incidence of IMD-attributable mortality—a particularly relevant concern in patients with hematological malignancies, in whom non-IMD deaths (e.g., due to relapse of underlying malignancy or chemotherapy-related complications) account for a substantial proportion of events. Our competing risk model showed that 36.0% of patients with mucormycosis died from IMD by week 12, providing a more accurate estimate of attributable mortality. Host immune status also significantly influences IMD prognosis. In our cohort, 32.1% of patients had neutropenia at IMD diagnosis (median duration 7 days), and 9.5% had prolonged neutropenia (>10 days). Consistent with the studies by Tuohuti et al. and Gandolpho et al. (19, 24), response of the underlying hematologic disease was an independent predictor of favorable clinical outcomes, underscoring the central role of controlling the underlying disease in the management of IMD.

### Safety profile

Isavuconazole demonstrated a favorable safety profile in our study, consistent with previous reports. Only 4.8% of patients experienced treatment-emergent adverse events, with one patient discontinuing therapy due to vomiting. Tuohuti et al. reported an adverse event rate of 33.3% but no treatment discontinuations (24), while Luo et al. reported an adverse event rate of 20.0% (18). The lower incidence of adverse events in our study may be related to incomplete recording of minor events inherent to the retrospective design. Ergün et al. specifically highlighted the clinical positioning of isavuconazole, showing that it remains a viable alternative even after toxicity with prior azole therapy (30). In our study, seven patients (8.3%) were switched to isavuconazole salvage therapy due to toxicity from prior antifungal agents, and none experienced further serious adverse events, supporting the value of isavuconazole in toxicity-driven switch therapy. Furthermore, the favorable renal safety profile of isavuconazole is particularly advantageous, making it a preferred option for patients with pre-existing renal impairment or those at high risk of nephrotoxicity.

### Limitations

Several limitations should be acknowledged. First, the retrospective design precludes causal inference and may introduce selection bias. Second, the relatively small sample size, particularly for the mucormycosis (*n* = 14) and mixed infection (*n* = 8) subgroups, limits the statistical power of subgroup analyses. Third, diagnosis was based on the EORTC/MSG criteria, with proven cases accounting for only 13.1% of the cohort; nevertheless, this reflects the diagnostic reality in patients with hematological malignancies, in whom invasive procedures are often contraindicated. Fourth, treatment decisions were not standardized, and the indications for combination therapy were heterogeneous. Moreover, the analyses that pool different infection types (aspergillosis, mucormycosis, and mixed infection) and different combination regimens are inherently limited; therefore, our subgroup comparisons should be considered exploratory rather than confirmatory. Fifth, although we collected data on neutropenia, we did not systematically assess the impact of neutrophil recovery timing on treatment outcomes. Sixth, therapeutic drug monitoring for isavuconazole was not routinely performed, precluding assessment of exposure-response relationships. Seventh, we did not capture the exact time interval from induction chemotherapy to IMD diagnosis, nor did we systematically assess the duration of neutropenia after IMD diagnosis or the timing of neutrophil recovery, which could provide additional insight into host immune status at infection onset and its impact on treatment outcomes. Finally, as a retrospective study, unmeasured confounding factors may exist.

### Clinical implications and future directions

Integrating our findings with the available literature, we propose the following clinical considerations. First, isavuconazole is an effective and well-tolerated treatment option, particularly for IA and as monotherapy. Its favorable safety profile makes it an ideal alternative for patients intolerant to prior azole therapy. Second, a stratified management strategy should be adopted for mucormycosis: isavuconazole is a suitable alternative to LAmB, especially for patients requiring prolonged oral therapy or those unable to tolerate LAmB. Third, combination therapy should be reserved for patients with severe or refractory disease, with the recognition that its use may reflect, rather than cause, poor outcomes. Fourth, alongside antifungal therapy, efforts should be made to control the underlying hematologic disease. Future prospective studies are needed to define optimal treatment strategies for mucormycosis, including the role of combination therapy and the timing of surgical intervention, as well as the value of therapeutic drug monitoring for individualized isavuconazole dosing. The mixed infection subgroup, though small, merits dedicated investigation in larger cohorts.

### Conclusions

In this two-center real-world study of 84 patients with hematological malignancies and IMD, isavuconazole achieved a week 6 treatment success rate of 59.5% with a favorable safety profile. Infection type and treatment strategy emerged as key prognostic factors: patients with IA and those receiving monotherapy experienced better outcomes, whereas mucormycosis and combination therapy were associated with significantly higher mortality risks. The high prevalence of neutropenia (32.1%) underscores the critical role of host immunity in antifungal treatment. These findings provide valuable evidence to guide antifungal optimization in this complex patient population.

## Data Availability

The data that support the findings of this study are available from the corresponding author upon reasonable request.

## SUPPLEMENTAL MATERIAL

Supplemental material aac.00543-26-s0001.docx Fig. S1; Tables S1 and S2.

ASM does not own the copyrights to Supplemental Material that may be linked to, or accessed through, an article. The authors have granted ASM a non-exclusive, world-wide license to publish the Supplemental Material files. Please contact the corresponding author directly for reuse.
