# Supplementary material for: Isavuconazole for invasive mold disease in patients with hematological malignancies: a multicenter real-world study from China on efficacy, safety, and competing risks
Source: Antimicrob Agents Chemother. 2026 Jul 6;70(8):e00543-26. doi: 10.1128/aac.00543-26 (PMC13436411; doi:10.1128/aac.00543-26)
Supplement: Supplementary file 1 [file aac.00543-26-s0001.docx]

**Supplementary Figure S1**

**Figure S1-A.** Kaplan‑Meier curves for week 12 all‑cause mortality stratified by therapy role (first‑line therapy, n=56; other indications, n=28). Tick marks indicate censored patients. Risk tables showing the number of patients at risk at weeks 0, 4, 8, and 12 are displayed below the curves. The log‑rank test showed no significant difference between the two groups (*P* = 0.568).

**Figure S1-B**. Kaplan‑Meier curves for week 12 all‑cause mortality stratified by treatment strategy (monotherapy, n=61; combination therapy, n=23). Tick marks indicate censored patients. Risk tables showing the number of patients at risk at weeks 0, 4, 8, and 12 are displayed below the curves. The log‑rank test showed no significant difference between the two groups (*P* = 0.069).

**Supplementary Table S1**

| **Patient ID** | **IMD type** | **Diagnostic level** | **GM serum**  **（＞0.5 ODs）;** | **GM BALF**  **（ ≥1.0 ODs）** | **Culture**  **(source; result)** | **Histopathology** | **mNGS**  **(source; result)** | **Summary of positive tests by EORTC/MSGERC 2020 criteria** |
| --- | --- | --- | --- | --- | --- | --- | --- | --- |
| 1 | Mixed | Probable | *–* | 2.6 (+) | *–* |  | BALF:Aspergillus fumigatus;Rhizomucor spp | GM BALF (+); mNGS (BALF) positive for Aspergillus and Mucorales |
| 2 | Aspergillosis | Probable | – | 2.91 (+) | BA：Aspergillus terreus |  | ND | GM BALF (+); culture (BA) positive for A. terreus |
| 3 | Aspergillosis | Probable | 0.55 (+) | ND | BALF：Aspergillus fumigatus |  | ND | GM serum (+); culture (BALF) positive |
| 4 | Aspergillosis | Probable | – | – | BA：Aspergillus fumigatus |  | ND | Culture (BA) positive |
| 5 | Aspergillosis | Proven | – | 4.61 (+) | pleural fluid：Aspergillus fumigatus |  | BALF:Aspergillus fumigatus | Proven by sterile-site culture (pleural fluid); also GM BALF (+) and mNGS (+) |
| 6 | Aspergillosis | Probable | – | – | *–* |  | BALF:Aspergillus flavus | mNGS (BALF) positive |
| 7 | Aspergillosis | Probable | – | ND | *–* |  | BALF:Aspergillus fumigatus | mNGS (BALF) positive |
| 8 | Aspergillosis | Proven | 0.77 (+) | 1.46 (+) | BA：Aspergillus fumigatus | Lung biopsy: septate hyphae consistent with Aspergillus spp. | BALF:Aspergillus fumigatus | Proven by histopathology; also GM serum/BALF (+), culture (+), mNGS (+) |
| 9 | Mucormycosis | Probable | *–* | *–* | *–* |  | BALF:Lichtheimia spp | mNGS (BALF) positive |
| 10 | Aspergillosis | Probable | – | 1.17 (+) | *–* |  | BALF:Aspergillus fumigatus | GM BALF (+); mNGS (+) |
| 11 | Aspergillosis | Probable | 5 | ND | *–* |  | Blood:Aspergillus flavus | GM serum (+); mNGS (blood) positive |
| 12 | Aspergillosis | Probable | – | – | *–* |  | BALF:Aspergillus fumigatus | mNGS (BALF) positive |
| 13 | Aspergillosis | Proven | 0.56 (+) | 4.99 (+) | BALF：Aspergillus fumigatus | Lung biopsy: fungal hyphae seen, morphology suggestive of Aspergillus | BALF:Aspergillus fumigatus | Proven by histopathology; also GM, culture, mNGS (+) |
| 14 | Aspergillosis | Probable | – | – | *–* |  | BALF:Aspergillus flavus | mNGS (BALF) positive |
| 15 | Aspergillosis | Probable | – | 4.09 (+) | BALF：Aspergillus fumigatus | *–* | – | GM BALF (+); culture (BALF) positive |
| 16 | Aspergillosis | Probable | – | – | BA：Aspergillus fumigatus；BALF：Aspergillus fumigatus |  | – | Culture (BA and BALF) positive |
| 17 | Aspergillosis | Probable | – | – | *–* |  | BALF:Aspergillus fumigatus | mNGS (BALF) positive |
| 18 | Mixed | Probable | 0.53 (+) | 2.4 (+) | *–* |  | BALF:Aspergillus fumigatus;Lichtheimia spp | GM serum (+), GM BALF (+); mNGS (BALF) dual positive |
| 19 | Aspergillosis | Probable | – | ND | BA：Aspergillus fumigatus |  | ND | Culture (BA) positive |
| 20 | Mucormycosis | Probable | *–* | *–* | *–* |  | BALF:Rhizomucor spp | mNGS (BALF) positive |
| 21 | Mixed | Probable | *–* | *–* | *–* |  | BALF:Aspergillus flavus;Lichtheimia spp | mNGS (BALF) dual positive |
| 22 | Aspergillosis | Probable | 0.68 (+) | ND | *–* |  | BALF:Aspergillus flavus | GM serum (+); mNGS (+) |
| 23 | Aspergillosis | Probable | – | – | BA：Aspergillus fumigatus |  | BALF:Aspergillus fumigatus | Culture (BA) positive; mNGS (+) |
| 24 | Aspergillosis | Probable | – | – | *–* |  | BALF:Aspergillus flavus | mNGS (BALF) positive |
| 25 | Aspergillosis | Probable | 4.39 (+) | 5.42 (+) | BA：Aspergillus flavus |  | – | GM serum (+), GM BALF (+); culture (BA) positive |
| 26 | Aspergillosis | Proven | 1.9 (+) | 2.7 (+) | *–* | Lung biopsy: septate hyphae consistent with Aspergillus spp. | BALF:Aspergillus fumigatus | Proven by histopathology; also GM (+), mNGS (+) |
| 27 | Aspergillosis | Probable | – | – | *–* |  | BALF:Aspergillus flavus | mNGS (BALF) positive |
| 28 | Mixed | Probable | *–* | ND | BA：Aspergillus fumigatus;Rhizomucor spp |  | BALF:Aspergillus fumigatus;Rhizomucor spp | Culture (BA) positive for Aspergillus and Rhizomucor; mNGS dual positive |
| 29 | Mucormycosis | Probable | *–* | *–* | *–* |  | BALF:Rhizopus spp | mNGS (BALF) positive |
| 30 | Aspergillosis | Probable | – | 1.23 (+) | BA：Aspergillus niger |  | – | GM BALF (+); culture (BA) positive for A. niger |
| 31 | Aspergillosis | Probable | – | 4.72 (+) | BA：Aspergillus sclerotiorum |  | – | GM BALF (+); culture (BA) positive for A. sclerotiorum |
| 32 | Aspergillosis | Probable | – | ND | BA：Aspergillus fumigatus |  | ND | Culture (BA) positive |
| 33 | Mucormycosis | Probable | 2.04 (+) | *–* | *–* |  | Blood:Lichtheimia spp | GM serum (+); mNGS (blood) positive |
| 34 | Aspergillosis | Probable | – | 1.72 (+) | BA:Aspergillus fumigatus |  | ND | GM BALF (+); culture (BA) positive |
| 35 | Aspergillosis | Probable | 0.71 (+) | 2.64 (+) | BALF：Aspergillus fumigatus |  | BALF:Aspergillus fumigatus | GM serum (+), GM BALF (+); culture (BALF) positive; mNGS (+) |
| 36 | Aspergillosis | Probable | – | 2.75 (+) | *–* |  | BALF:Aspergillus niger | GM BALF (+); mNGS (BALF) positive for A. niger |
| 37 | Mixed | Probable | *–* | 3.3 (+) | BA：Aspergillus fumigatus;Rhizomucor spp |  | BALF:Aspergillus fumigatus;Rhizomucor spp | Culture (BA) and mNGS (BALF) dual positive for Aspergillus and Rhizomucor |
| 38 | Aspergillosis | Proven | 5.48 (+) | 5.12 (+) | *–* | Lung biopsy: fungal hyphae seen, morphology suggestive of Aspergillus | BA:Aspergillus fumigatus | Proven by histopathology; also GM (+), culture (+) |
| 39 | Aspergillosis | Proven | 3.22 (+) | 4.33 (+) | BA：Aspergillus fumigatus；BALF：Aspergillus fumigatus；pleural fluid：Aspergillus fumigatus |  | ND | Proven by sterile-site culture (pleural fluid); also culture (BA, BALF) positive, GM (+) |
| 40 | Mucormycosis | Probable | *–* | 1.34 (+) | BA：Lichtheimia spp |  | BALF:Lichtheimia spp | GM BALF (+); culture (BA) positive for Lichtheimia; mNGS (+) |
| 41 | Aspergillosis | Probable | – | 1.29 (+) | *–* |  | BALF:Aspergillus flavus | GM BALF (+); mNGS (BALF) positive |
| 42 | Mixed | Probable | *–* | *–* | BA：Aspergillus niger |  | BALF:Aspergillus niger;Rhizopus spp | Culture (BA) positive for A. niger; mNGS (BALF) dual positive |
| 43 | Aspergillosis | Probable | – | – | *–* |  | Blood:Aspergillus fumigatus | mNGS (blood) positive |
| 44 | Aspergillosis | Probable | 0.71 (+) | 6.92 (+) | BALF：Aspergillus niger |  | ND | GM serum (+), GM BALF (+); culture (BALF) positive |
| 45 | Mixed | Probable | 0.55 (+) | 5.09 (+) | *–* |  | BALF:Aspergillus fumigatus;Rhizomucor spp | GM serum (+), GM BALF (+); mNGS (BALF) dual positive |
| 46 | Mucormycosis | Probable | *–* | *–* | *–* |  | BALF:Lichtheimia spp | mNGS (BALF) positive |
| 47 | Aspergillosis | Probable | ND | ND | BA:Aspergillus fumigatus |  | BALF:Aspergillus fumigatus | Culture (BA) positive; mNGS (BALF) positive |
| 48 | Aspergillosis | Probable | – | – | *–* |  | BALF:Aspergillus fumigatus | mNGS (BALF) positive |
| 49 | Mixed | Probable | *–* | ND | *–* |  | BALF:Aspergillus flavus;Rhizomucor spp | mNGS (BALF) dual positive |
| 50 | Aspergillosis | Probable | – | ND | *–* |  | BALF:Aspergillus flavus | mNGS (BALF) positive |
| 51 | Aspergillosis | Probable | – | ND | *–* |  | Blood:Aspergillus glaucus | mNGS (blood) positive |
| 52 | Aspergillosis | Probable | – | *–* | *–* |  | BALF:Aspergillus fumigatus | mNGS (BALF) positive |
| 53 | Mucormycosis | Probable | *–* | ND | *–* |  | Blood:Rhizomucor spp | mNGS (blood) positive |
| 54 | Aspergillosis | Probable | – | ND | *–* |  | BALF:Aspergillus fumigatus | mNGS (BALF) positive |
| 55 | Aspergillosis | Probable | – | ND | BA：Aspergillus fumigatus |  | BA:Aspergillus fumigatus | Culture (BA) positive; mNGS (BA) positive |
| 56 | Mucormycosis | Probable | *–* | ND | *–* |  | Blood:Cunninghamella spp | mNGS (blood) positive |
| 57 | Aspergillosis | Probable | – | ND | *–* |  | BALF:Aspergillus fumigatus | mNGS (BALF) positive |
| 58 | Aspergillosis | Probable | – | ND | *–* |  | BALF:Aspergillus fumigatus | mNGS (BALF) positive |
| 59 | Aspergillosis | Probable | – | ND | *–* |  | BALF:Aspergillus fumigatus | mNGS (BALF) positive |
| 60 | Aspergillosis | Probable | – | *–* | *–* |  | BALF:Aspergillus fumigatus | mNGS (BALF) positive |
| 61 | Mucormycosis | Probable | *–* | ND | *–* |  | Blood:Cunninghamella spp | mNGS (blood) positive |
| 62 | Aspergillosis | Probable | – | ND | *–* |  | Blood:Aspergillus flavus | mNGS (blood) positive |
| 63 | Aspergillosis | Probable | – | ND | BA：Aspergillus fumigatus |  | ND | Culture (BA) positive |
| 64 | Aspergillosis | Probable | – | ND | ND |  | BALF:Aspergillus niger | mNGS (BALF) positive |
| 65 | Mucormycosis | Probable | *–* | ND | *–* |  | Blood:Rhizopus spp | mNGS (blood) positive |
| 66 | Aspergillosis | Probable | – | ND | BA：Aspergillus fumigatus |  | ND | Culture (BA) positive |
| 67 | Aspergillosis | Probable | – | ND | BA：Aspergillus fumigatus |  | ND | Culture (BA) positive |
| 68 | Aspergillosis | Probable | – | ND | BA：Aspergillus fumigatus |  | ND | Culture (BA) positive |
| 69 | Aspergillosis | Probable | – | ND | BA：Aspergillus fumigatus |  | ND | Culture (BA) positive |
| 70 | Mucormycosis | Proven | *–* | ND | BA：Lichtheimia spp. | Lung biopsy: fungal hyphae consistent with Mucorales | BALF:Lichtheimia spp | Culture (BA) positive for Lichtheimia; mNGS (BALF) positive；Proven by histopathology (Mucorales) |
| 71 | Aspergillosis | Probable | – | ND | BA：Aspergillus fumigatus |  | ND | Culture (BA) positive |
| 72 | Mucormycosis | Probable | 8.44 (+) | ND | BA: Rhizomucor spp |  | BALF:Rhizomucor spp | GM serum (+); culture (BA) positive for Rhizomucor; mNGS (BALF) positive |
| 73 | Aspergillosis | Probable | 2.16 (+) | ND | BA：Aspergillus fumigatus |  | ND | GM serum (+); culture (BA) positive |
| 74 | Aspergillosis | Probable | – | – | *–* |  | BALF:Aspergillus fumigatus | mNGS (BALF) positive |
| 75 | Aspergillosis | Probable | – | ND | BA：Aspergillus fumigatus |  | BALF:Aspergillus fumigatus | Culture (BA) positive; mNGS (BALF) positive |
| 76 | Aspergillosis | Probable | – | ND | ND |  | BALF:Aspergillus flavus | mNGS (BALF) positive |
| 77 | Aspergillosis | Probable | – | – | *–* |  | BALF:Aspergillus fumigatus | mNGS (BALF) positive |
| 78 | Aspergillosis | Probable | – | ND | BA：Aspergillus fumigatus |  | ND | Culture (BA) positive |
| 79 | Aspergillosis | Probable | – | – | *–* |  | BALF:Aspergillus niger | mNGS (BALF) positive |
| 80 | Mucormycosis | Probable | *–* | *–* | *–* |  | BALF:Rhizomucor spp | mNGS (BALF) positive |
| 81 | Aspergillosis | Proven | 2.35 (+) | 5.72 (+) | pleural fluid: Aspergillus fumigatus;BA:Aspergillus fumigatus;BALF:Aspergillus fumigatus |  | ND | Proven by sterile-site culture (pleural fluid); also culture (BA, BALF) positive, GM (+) |
| 82 | Aspergillosis | Probable | 2.2 (+) | ND | BA：Aspergillus fumigatus |  | ND | GM serum (+); culture (BA) positive |
| 83 | Aspergillosis | Probable | 2.4 (+) | ND | BA：Aspergillus fumigatus |  | ND | GM serum (+); culture (BA) positive |
| 84 | Mucormycosis | Proven | 5.5 (+) | ND | BA: Rhizomucor spp | Lung biopsy: broad, aseptate hyphae with right‑angle branching, consistent with Mucorales infection | Blood:Rhizomucor spp | GM serum (+); culture (BA) positive for Rhizomucor; mNGS (blood) positive；Proven by histopathology (consistent with Mucorales) |

**Supplementary Table S1. Patient‑level microbiological test results for 84 patients with IMD (proven/probable).**

GM (positivity: serum ≥0.5, BALF ≥1.0), culture, histopathology, and mNGS results are shown for each patient (abbreviations: BA, bronchial aspirate; BALF, bronchoalveolar lavage fluid; ND, not done). Symbols: + (GM) positive with index value; – negative. Proven cases: decisive evidence (histopathology or sterile‑site culture) is highlighted in the last column. mNGS results are supportive (not yet formally in EORTC/MSGERC criteria). One patient may have multiple positive tests; the summary column integrates all relevant findings.

**Supplementary Table S2**

| Comparison | Log‑rank *P* value |
| --- | --- |
| *Aspergillosis* vs. mucormycosis | 0.027 |
| *Aspergillosis* vs. mixed infection | 0.47 |
| Mucormycosis vs. mixed infection | 0.58 |

**Supplementary Table S2**. Pairwise comparisons of week‑12 all‑cause mortality among the three infection types (invasive aspergillosis [IA], mucormycosis, and mixed infection). *P* values were calculated using the log‑rank test. The overall log‑rank test across all three groups was not significant (*P* = 0.087), as reported in the main manuscript (Figure 4).
